# Maintenance of chronicity signatures in fibroblasts isolated from recessive dystrophic epidermolysis bullosa chronic wound dressings under culture conditions

**DOI:** 10.1186/s40659-023-00437-2

**Published:** 2023-05-10

**Authors:** Cristian De Gregorio, Evelyng Catalán, Gabriel Garrido, Pilar Morandé, Jimena Castillo Bennett, Catalina Muñoz, Glenda Cofré, Ya-Lin Huang, Bárbara Cuadra, Paola Murgas, Margarita Calvo, Fernando Altermatt, María Joao Yubero, Francis Palisson, Andrew P. South, Marcelo Ezquer, Ignacia Fuentes

**Affiliations:** 1 Centro de Medicina Regenerativa, Facultad de Medicina Clínica Alemana, Universidad del Desarrollo, Santiago, 7610658 Chile; 2 DEBRA Chile, Francisco de Villagra 392, Ñuñoa, Santiago, Chile; 3 Instituto de Bioquímica y Microbiología, Facultad de Ciencias, Universidad Austral de Chile, Valdivia, Chile; 4 Facultad de Ciencias Biológicas y División de Anestesiología, Escuela de Medicina, Pontificia Universidad Católica de Chile, Santiago, Chile; 5 Núcleo milenio para el estudio del dolor MINUSPAIN, Santiago, Chile; 6 División de Anestesiología, Escuela de Medicina, Pontificia Universidad Católica de Chile, Santiago, Chile; 7 Pediatrics and Pediatric Infectious Diseases of Clínica Alemana, Facultad de Medicina Alemana, Universidad del Desarrollo, Santiago, Chile; 8 Servicio de Dermatología, Facultad de Medicina Clínica Alemana, Universidad del Desarrollo, Santiago, Chile; 9 Department of Dermatology & Cutaneous Biology, Thomas Jefferson University, Philadelphia, USA; 10 Centro de Genética y Genómica, Facultad de Medicina Clínica Alemana, Universidad del Desarrollo, Santiago, 7610658 Chile; 11 Departamento de Biología Celular y Molecular, Facultad de Ciencias Biológicas, Pontificia Universidad Católica de Chile, Santiago, Chile

**Keywords:** Recessive Dystrophic Epidermolysis Bullosa, Skin fibroblast, Chronic Wounds, Wound Dressing, Fibrosis

## Abstract

**Background:**

Recessive Dystrophic Epidermolysis Bullosa (RDEB) is a rare inherited skin disease caused by variants in the *COL7A1* gene, coding for type VII collagen (C7), an important component of anchoring fibrils in the basement membrane of the epidermis. RDEB patients suffer from skin fragility starting with blister formation and evolving into chronic wounds, inflammation and skin fibrosis, with a high risk of developing aggressive skin carcinomas. Restricted therapeutic options are limited by the lack of in vitro models of defective wound healing in RDEB patients.

**Results:**

In order to explore a more efficient, non-invasive in vitro model for RDEB studies, we obtained patient fibroblasts derived from discarded dressings) and examined their phenotypic features compared with fibroblasts derived from non-injured skin of RDEB and healthy-donor skin biopsies. Our results demonstrate that fibroblasts derived from RDEB chronic wounds (RDEB-CW) displayed characteristics of senescent cells, increased myofibroblast differentiation, and augmented levels of TGF-β1 signaling components compared to fibroblasts derived from RDEB acute wounds and unaffected RDEB skin as well as skin from healthy-donors. Furthermore, RDEB-CW fibroblasts exhibited an increased pattern of inflammatory cytokine secretion (IL-1β and IL-6) when compared with RDEB and control fibroblasts. Interestingly, these aberrant patterns were found specifically in RDEB-CW fibroblasts independent of the culturing method, since fibroblasts obtained from dressing of acute wounds displayed a phenotype more similar to fibroblasts obtained from RDEB normal skin biopsies.

**Conclusions:**

Our results show that in vitro cultured RDEB-CW fibroblasts maintain distinctive cellular and molecular characteristics resembling the inflammatory and fibrotic microenvironment observed in RDEB patients’ chronic wounds. This work describes a novel, non-invasive and painless strategy to obtain human fibroblasts chronically subjected to an inflammatory and fibrotic environment, supporting their use as an accessible model for in vitro studies of RDEB wound healing pathogenesis. As such, this approach is well suited to testing new therapeutic strategies under controlled laboratory conditions.

**Supplementary Information:**

The online version contains supplementary material available at 10.1186/s40659-023-00437-2.

## Background

Epidermolysis Bullosa (EB) is a group of genodermatoses characterized by skin and mucosa fragility and the formation of blisters due to mechanical stress [1]. EB, while varied in presentation and severity, is caused by mutations in genes coding for proteins that maintain the normal structural integrity of skin. Patients with EB are classified into four general types based on the genetic defects and the area of the skin where fragility is most evident: EB simplex (EBS); Junctional EB (JEB); Dystrophic EB (DEB); and Kindler EB (KEB) [2, 3].

The four general types of EB are further divided into specific sub-types which have their own clinical characteristics and disease severity, some being more incapacitating than others [3]. One of the more debilitating sub-types of EB is Recessive Dystrophic EB (RDEB), which itself can be sub-classified into RDEB intermediate and RDEB severe. Each RDEB subtypes is caused by recessive variants in the *COL7A1* gene and have a highly impaired or null accumulation of type VII collagen protein (C7) [4]. As a consequence of this genetic defect, patients have fragile skin and minimal trauma can trigger painful blisters and wounds that are often very slow to heal. Moreover, patients with RDEB have poor clinical prognosis and a high risk of developing aggressive cutaneous squamous cell carcinoma (cSCC) early in life [4–6].

The cancer-prone stromal microenvironment observed in RDEB is influenced by chronic inflammation as well as fibrosis [7–10]. A major factor contributing to fibrosis in RDEB are isoforms of Transforming Growth Factor-β (TGF-β), primarily TGF-β1. In a normal wound healing process, this factor participates in the differentiation of fibroblasts into myofibroblasts, characterized by the increased production of alpha-smooth muscle actin (α-SMA) and the formation of stress fibers, which help to mechanically close wounds by contraction [11]. However, in the context of impaired healing in RDEB, wounds and skin experience chronic fibrosis which leads to stiffening of the extracellular matrix (ECM) and release of latent TGF-β1. The latter generates a positive feedback loop of fibrosis and inflammation, as TGFβ-induced myofibroblast differentiation is not only associated with altered ECM deposition, but also to increased production of multiple inflammatory cytokines [4].

Blisters and wounds in RDEB have been characterized as being highly inflamed, with several inflammatory cytokines identified as increased in the scar tissue and serum of patients [12–14]. Of interest among these inflammatory cytokines is interleukin-6 (IL-6), a cytokine that normally plays a role in proper wound closure but has also been associated with more severe manifestations of RDEB [15]. IL-6 accumulation induces interleukin-1β (IL-1β) production in macrophages, which in turn induces keratinocyte growth factor (KGF) production in fibroblasts. This allows the proliferation and migration of keratinocytes, thus stimulating wound re-epithelization and closure [15, 16]. Moreover, IL-6 also induces VEGF expression in macrophages, fibroblasts, and keratinocytes, which allows for the vascularization of the repaired tissue [14, 17]. However, a prolonged inflammatory microenvironment in a wound, increases leukocyte and matrix metalloproteinase activity, hence, impairing the healing process. The transition from an inflammatory state to a proliferative state plays a crucial role in determining the outcome of wound closure, and chronic wounds are associated with an inability to overcome the inflammatory phase of wound healing [18–20].

Most researchers studying EB physiopathology utilize patient’s serum [14, 21] or cells grown from tissue biopsies [13]. Although many articles have shown that these samples successfully recreate some of the molecular and physiological parameters observed in RDEB [22–26], there are currently no efficient models to study this disease in vitro in the context of chronic wounds. The latter is a consequence of RDEB patients suffering from extreme skin fragility and neuropathic pain [27–29], restricting the availability of skin or wound biopsies for research purposes. Hence, there is an urgent need to identify models that allow a more accurate representation of RDEB wound healing in vitro.

Recent work of ours has shown that RDEB wound dressings contain viable host cells that can be utilized for in vitro studies [30]. Understanding whether this method of sample collection can differentiate primary cells derived from a pro-inflammatory and pro-fibrotic microenvironment during repeated cycles of wounding and healing will provide a non-invasive and relatively simple approach to study RDEB. In this report, we characterize and further explore the use of adherent cells isolated from RDEB chronic wound dressings as an in vitro model of wound chronicity.

## Results

### General characterization of cells isolated from RDEB patient’s chronic wound dressings

**Fig. 1:**
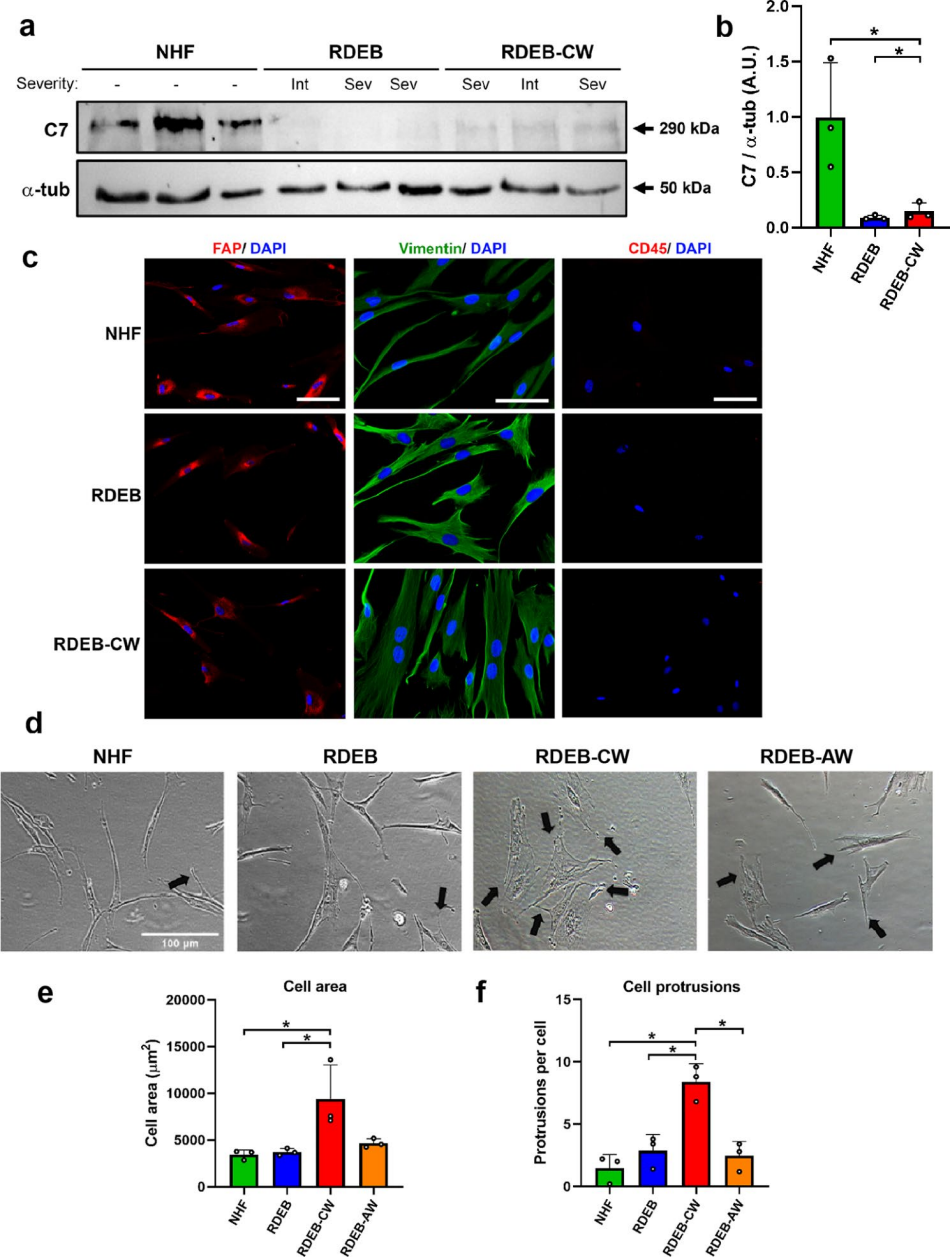
**General phenotypic characterization of RDEB-CW cells. (a)** Western blot of protein lysates derived from fibroblast cultures confirms the C7 deficiency in all samples coming from RDEB patients used in this study, with its respective densitometric analysis in **(b)**. **(c)** Representative IIF images showing molecular markers for fibroblasts (FAP, vimentin) and white blood cells (CD45). Nuclei were co-stained with DAPI. Bar: 100 μm. **(d-f)** Representative bright field images of NHFs, RDEBs, RDEB-CWs and RDEB-AWs fibroblast cultures used for morphological evaluation. Black arrows indicate cell protrusions. The morphological parameters analyzed were cell area **(e)** and the mean protrusions per cell **(f)**. For the quantitative analysis in **(e-f)**, a total of 75 cells were used for each condition (225 cells per experimental group). Bar: 100 μm. All data are expressed as mean ± SD. All these experiments were performed with n = 3–4 per condition. Asterisks indicate significant differences by one-way ANOVA with Tukey post-hoc (p < 0.05)

In this study, we used adherent cells with fibroblast-like morphology isolated from discarded dressings coming from chronic RDEB patients’ wounds, previously described in [30] and referred here as to RDEB-CW hereon (see Materials and Methods). Chronic wounds were defined as wounds that were open for more than 1 year at the time of dressing sampling, and remained open for at least 3 months after the sampling date. In order to characterize these cells and evaluate their potential to become an in vitro model of RDEB wound healing, we compared them with fibroblasts derived from skin biopsies, either from RDEB patients or from healthy donors. These fibroblasts were used as controls and are referred as to RDEB fibroblasts or normal human fibroblasts (NHF), respectively. Altogether, there were 6 samples from RDEB-CW from 3 patients, 3 samples of RDEB-AW from 2 patients, 4 skin biopsies from unaffected RDEB skin, and 7 samples from normal skin (Table 1**).** We did not obtain paired samples between the RDEB, RDEB-AW, and RDEB-CW groups. Western blot analysis (WB) confirmed the strong reduction of C7 protein in all RDEB samples (Fig. 1a, b), either coming from biopsies or wounds, as expected for samples derived from RDEB patients.

**Table 1:** General information of RDEB patients and healthy donors participating in this study

Sample Name	Age		Sex		RDEB subtype		Sample type		Sample code		Body location		Mutations		
SA01F		37		F		-		Skin biopsy		NHF		Abdomen		-	
SA02F		38		F		-		Skin biopsy		NHF		Abdomen		-	
SA03F		39		F		-		Skin biopsy		NHF		Abdomen		-	
SA04F		49		F		-		Skin biopsy		NHF		Abdomen		-	
SA05F		49		F		-		Skin biopsy		NHF		Abdomen		-	
Br1		N/A		F		-		Skin biopsy		NHF		Breast		-	
Br2		N/A		F		-		Skin biopsy		NHF		Breast		-	
RDEB1-01 month 3		3		F		Gen. Int.		Wound		RDEB-CW		Right heel		c.6527insC/ c.6527insC	
RDEB1-02 month 3												Left ankle			
RDEB1-20 month 3										RDEB-AW		Right elbow			
pEB56F		19		M		Gen. Int.		Skin biopsy		RDEB		Right arm		c.6527insC /c.-185 C > T	
pEB111F		11		F		Gen. Int.		Skin biopsy		RDEB		Left arm		c.6527insC/ c.8329 C > T	
pEB210F		18		F		Gen. Severe		Skin biopsy		RDEB		Ankle		c.7708delG/ c.7708delG	
RDEB4-03 month 0		20		M		Gen. Severe		Wound		RDEB-AW		Right leg		c.6527insC/ c.6527insC	
RDEB4-04 month 0												Right ankle			
RDEB6-01 month 3 RDEB6-02 month 3 RDEB6-01 month 6		23		F		Gen. Severe		Wound		RDEB-CW		Left arm Left flank Left arm		c.6527insC/ c.6527insC	
pEB69F		25						Skin biopsy		RDEB		Ankle			
RDEB8-01 month 0		24		F		Gen. Severe		Wound		RDEB-CW		Lumbar region		c.6527insC/ c.7708delG	

The isolation of cells from discarded wound dressings allows the enrichment of adherent cells in culture. Different populations of both adherent and non-adherent cells can be extracted from these dressings, such as fibroblasts, corneocytes, immune cells, mesenchymal stem cells (MSC), fibrocytes, among others [30]. To characterize the cell lineage from which RDEB-CW adherent cells were derived, we utilized different cell markers [31–33] and analyzed their expression profiles by indirect immunofluorescence (IIF). All six separate RDEB-CW samples were composed of adherent cells showing a positive signal for fibroblast activating protein (FAP, a type II serine protease expressed in fibroblasts and not detected on fibrocytes) [31] and vimentin, both being common markers distinguishing fibroblasts (Fig. 1c) [34]. On the other hand, these cells show no expression of CD45 protein, a common antigen expressed in white blood cells, as well as fibrocytes [31, 32] (Fig. 1c). Positive controls for CD45 (THP-1 cells) and negative controls for all the used markers are depicted in Supplementary Fig. 1a and b. Taken together, all these analyses indicate that RDEB-CW adherent cells correspond to a cell lineage consistent primarily with being fibroblasts, thus, from now on these cells will be referred in the text as RDEB-CW fibroblasts.

In culture, RDEB-CW fibroblasts exhibited an enlarged cell area when compared to NHF and RDEB fibroblasts isolated from uninvolved skin (Fig. 1d, e). Also, in contrast to the normal spindle-like morphology of fibroblasts, RDEB-CW displayed an increased number of cell protrusions (Fig. 1f), suggesting an activated state of these fibroblasts [35]. The latter indicates that RDEB-CW fibroblasts maintain a chronicity-associated phenotype even when cultured in vitro under controlled conditions.

In order to determine whether the distinct RDEB-CW phenotype observed in culture results from the method of isolation, we compared RDEB-CW fibroblasts with fibroblasts derived from acute wounds (RDEB-AW, wounds than were opened for less than 8 weeks, see Materials and Methods). We showed that RDEB-AW fibroblasts displayed a phenotype similar to fibroblasts derived from RDEB patients skin biopsies (Fig. 1, d-f), suggesting that the cell morphology differences observed in RDEB-CW fibroblasts are not due to the cell harvesting method.

### Functional analyses in RDEB-CW fibroblasts

**Fig. 2:**
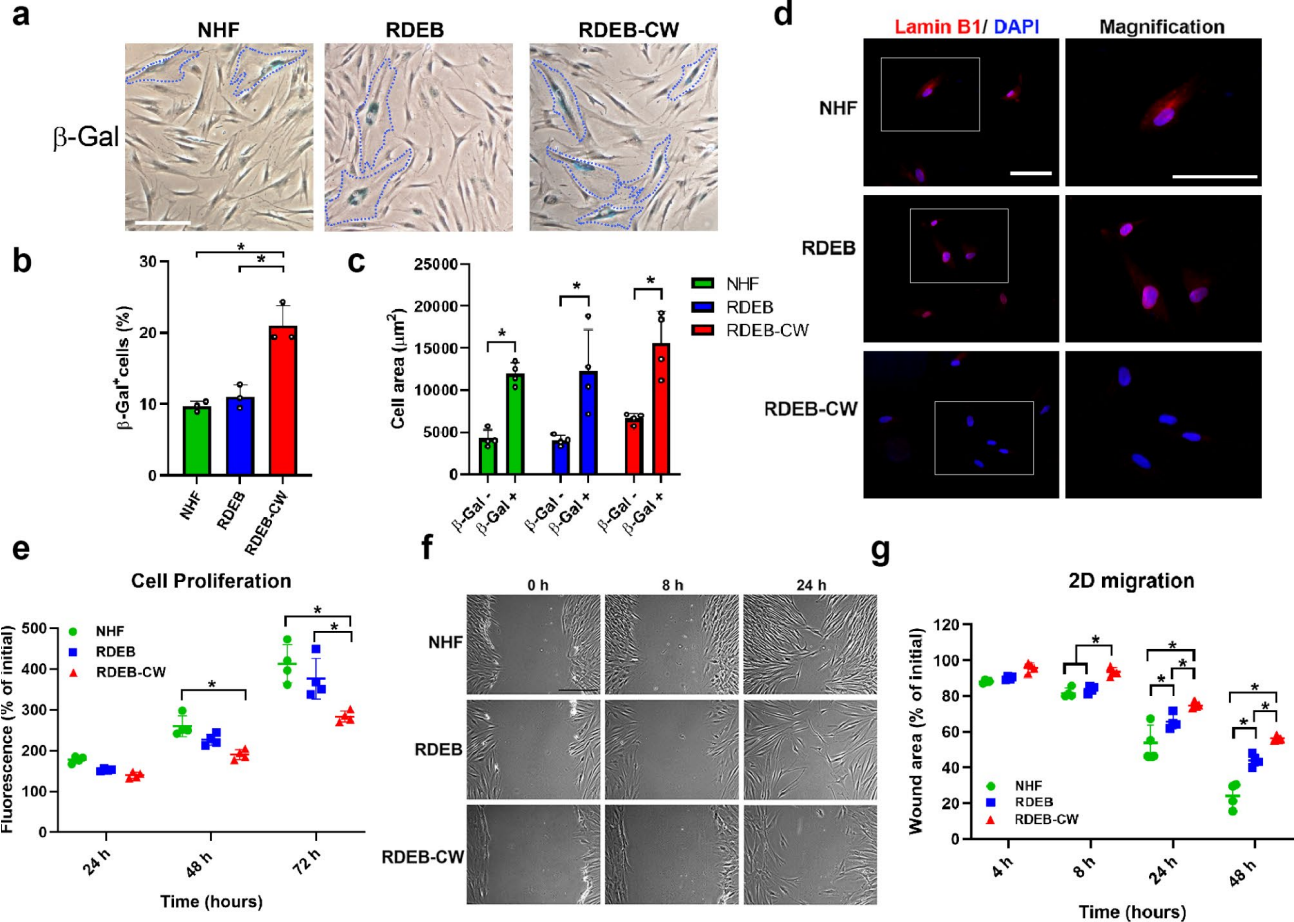
**Functional characterization in RDEB-CW fibroblasts. (a-c)** Representative bright field images of fibroblast cultures stained with the senescence associated β-galactosidase marker. β-Gal + cells were delimited with a dashed blue line. The quantification of β-Gal + cell population and the measurement of the cell area for β-Gal + and β-Gal- cells are depicted in **(b)** and **(c)**, respectively. For the quantitative analysis in **(b)**, a total of ~ 4000 cells were used per condition. For the quantitative analysis in **(c)**, a total of 50 cells were used for each condition (150 cells per experimental group). Bar: 100 μm. **(d)** Representative fluorescence images of fibroblast cultures stained with Lamin B1 (red), and counterstained with DAPI (blue). Bar: 100 μm.  **(e)** Fluorescence-based proliferation assay (Cell Titer Blue ®) assessed every 24 h during 72 h.  **(f)** Representative brightfield images of 2D migration assay. Bar: 100 μm. **(g)** Quantification of migration rate, expressed as percentage of the initial wound area. All data are expressed as mean ± SD. All these experiments were performed with n = 3–4 per condition. Asterisks indicate significant differences by one-way ANOVA with Tukey post-hoc (p < 0.05)

Flat and enlarged cell morphology, and an increased number of cell protrusions are well described features of senescent fibroblasts [35, 36]. Thus, we assessed whether RDEB-CW fibroblasts exhibit features of a senescent phenotype. β-galactosidase staining is a widely used marker for measuring cellular senescence both in vitro and in vivo [36, 37]. Our results indicate that NHF and RDEB fibroblasts displayed senescent populations of around 10%, which is in agreement with previous observations (Fig. 2a, b) [36–38]. In contrast, RDEB-CW fibroblasts exhibited a 2-fold increase in senescent cells compared to NHF and RDEB fibroblasts. In line with the previous results, we observed that senescent fibroblasts (β-gal^+^ cells) exhibited an increased cell area compared to β-gal^-^ cells, independent of the fibroblast culture condition analyzed (Fig. 2c). We did not observe significant differences between groups when we compared the β-gal^+^ cells or the β-gal^-^ cells, suggesting that the morphology differences associated to RDEB-CW fibroblasts is likely due to an increased senescent population.

Lamin B1 protein has also been studied as a marker for cell senescence, where the loss of its nuclear localization is correlated with cells entering a senescent phase [39]. Interestingly, we found a loss of the nuclear localization of LaminB1 in RDEB-CW fibroblasts (Fig. 2d and Supplementary Fig. 2). Taken together, these results suggest that repeated cycles of wounding and healing associated to chronic wounds could affect the senescent status of fibroblast populations isolated from these wounds, and that this phenotype can be further maintained under in vitro culture conditions.

Population doublings is an important factor contributing to cell senescence under in vitro conditions [40, 41]. Thus, an obvious consideration when comparing cell morphologies from different sources is the number of doublings in culture. In our study, all cells investigated were coming from low culture passages. However, to exclude the possibility that RDEB-CW fibroblasts became senescent during cell isolation and culturing, we compared the number of viable cells initially obtained from chronic and acute wound dressings. No difference in number of cells (adjusted to wound size) were observed between RDEB-AW and RDEB-CW cells (Supplementary Fig. 3), suggesting that the senescent phenotype of RDEB-CW fibroblasts is not a consequence of the isolation and cell culture procedure.

After wounding, dermal fibroblasts actively start proliferating and migrating into the wound bed, constituting a key event in wound healing [42]. Thus, we investigated whether these processes were affected in RDEB-CW fibroblasts in vitro. Our results indicated that RDEB-CW fibroblasts displayed a reduced proliferative rate compared to NHF by a Cell Titer-Blue® assay, meanwhile RDEB fibroblasts showed no significant differences compared to the control group (Fig. 2e). These results were complemented by an IIF analysis, using the proliferation marker Proliferating cell nuclear antigen (PCNA), where we found similar results to those obtained by the fluorometric technique (Supplementary Fig. 4a, b). Furthermore, RDEB-CW fibroblasts displayed slower wound closure compared to NHF (Fig. 2f-g). RDEB fibroblasts also exhibited a mild, but significant, decrease in migration potential. Similar results were obtained when this assay was carried out in low-serum condition (0.5% fetal bovine serum) and using an anti-proliferative agent (5 µM Cytosine β-D-arabinofuranoside, ARAC), indicating that these defects are not affected by the different proliferative rates observed between the three experimental groups (Supplementary Fig. 5).

### Fibrotic marker assessment of RDEB-CW fibroblasts

A decrease or complete absence of C7 is the primary deficit of RDEB patients and it has been associated with an impaired remodeling and deposition of ECM proteins, triggering the development of a profibrotic environment on RDEB patient skin and wounds. Furthermore, increased levels of ECM proteins have been related to disease severity and a high risk for developing cSCCs, the primary cause of death in this patient population [5, 43–48]. With this information in mind and our previous results, we hypothesized that RDEB-CW fibroblasts exhibit a unique phenotype under in vitro culturing conditions whichx| could be used as a model to study the development of fibrosis and resulting pathologies.

**Fig. 3:**
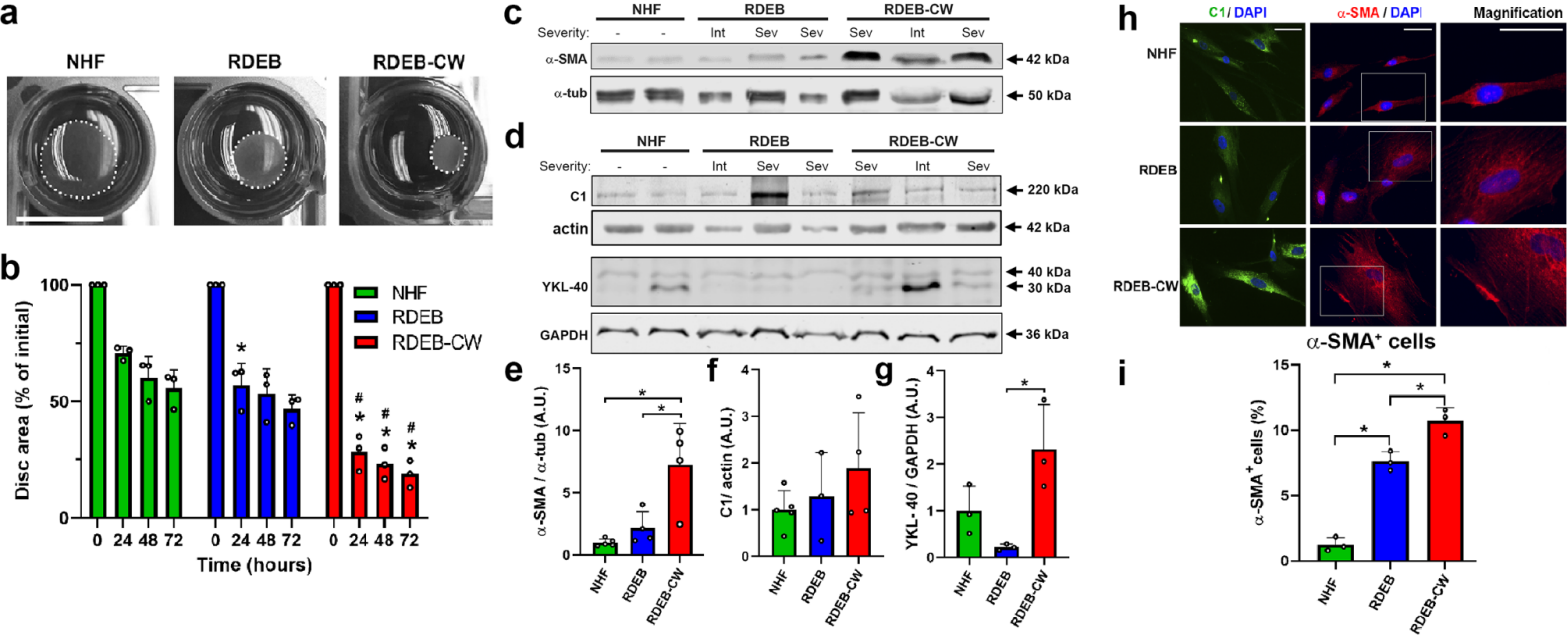
**Functional and molecular markers of fibrosis in RDEB-CW fibroblasts**. **(a)** Representative images of contracted collagen discs after 24 h of in vitro culture. The size decreased as the contractile capability of the fibroblasts increased. Bar: 1 cm. **(b)** Quantification of contractile assay based on collagen lattices (n = 3). We carried out a statistical analysis to compare the disc contraction between the different experimental groups at 0, 24, 48, and 72 h. Asterisks (*) indicate significant differences versus NHF. Hashtags (#) indicate significant differences versus the RDEB group (p < 0.05, One-way ANOVA with Tukey post-hoc). **(c-d)** Western blot analysis of protein lysates derived from fibroblast cultures to detect the fibrotic markers α-SMA, C1 and YKL-40. **(e-g)** Densitometric analysis of western blots displayed in **(c-d).** Asterisks indicate significant differences (n = 3, one-way ANOVA with Tukey post-hoc). **(h)** IIF analysis of Collagen I (green) and α-SMA (red). Nuclei were co-stained with DAPI (blue). Bar: 100 μm. **(i)** Quantification of α-SMA^+^ cells, expressed as percentage. Fibroblasts were considered as positives for α-SMA specifically when this protein was localized to stress fibers (n = 3). Asterisks indicate significant differences (p < 0.05, One-way ANOVA with Tukey post-hoc). All data are expressed as mean ± SD.

Fibrotic fibroblasts have an increased capacity to secrete and interact with ECM proteins, and are known to have an enhanced contractile phenotype in a three-dimensional fibrous collagen network [25, 27]. Hence, we decided to study the fibrotic potential of RDEB-CW fibroblasts. We carried out a collagen disc contraction assay, where we demonstrated the increased contractile behavior of RDEB-CW cells (Fig. 3a, b). As control, we included RDEB fibroblasts which have been shown to possess an enhanced ability to contract collagen lattices under in vitro conditions compared to NHF [49, 50]. Interestingly, RDEB-CW fibroblasts contractility was greater than RDEB fibroblasts at all evaluated times.

Next, we studied the expression of fibrotic biomarkers previously shown to be increased in RDEB skin, such as alpha-smooth muscle actin (α-SMA) and type I collagen (C1) [47]. α-SMA, a protein associated with fibroblast-myofibroblast transition and fibroblast activation [51], was increased at protein level (Fig. 3c, e) and enriched in stress fibers in RDEB-CW fibroblasts (Fig. 3h, i), where it is associated with actin filaments to exert its contractile functions [52].

Interestingly, no differences were observed in C1 protein abundance in RDEB-CW fibroblasts when studied by western blot analysis (Fig. 3d, f). However, a noticeable increase in cytoplasmatic accumulation of this protein was seen by indirect immunofluorescence (Fig. 3h**).** We also assessed the expression of YKL-40 (Also known as CHI3L1), a collagen-binding glycoprotein mainly produced by cells of immune system as macrophages, neutrophils and cancer cells [53]. Fibroblasts can sense this protein and use it as a growth factor improving cell proliferation, survival, cell-matrix interactions and deposition of ECM during wound healing [54–56]. Recently, Scotece et al. demonstrated that YKL-40 exhibited a sharp increase in skin in response to bleomycin treatment [57], and it has also been previously related to the progression of several inflammatory and fibrotic skin diseases, including systemic sclerosis, dermatitis and pyoderma gangrenosum [58–60]. Additionally, Ho et al. (2014) demonstrated that a reduced population of primary dermal cells in culture (5–10% of total population) expresses YKL-40, but the expression was more widespread when the cells were treated with oncostatin M, a cytokine related to IL-6 family [61]. Our results showed a significant increase in YKL-40 protein level in RDEB-CW fibroblasts compared with RDEB fibroblasts, which supports the advanced fibrotic state of the cells derived from chronic wounds (Fig. 3d, g).

### TGF-β pathway signaling assessment in RDEB-CW fibroblasts

As mentioned previously, there is a positive feedback loop between fibrosis and inflammation, which is especially deleterious in RDEB. Several studies have shown the dysregulation of TGF-β1 expression and downstream related proteins in cells derived from RDEB patients [47, 49, 62]. Moreover, the activation of this TGF- β1 pathway has been related to correlate with disease severity [63]. Due to this, we decided to further explore TGF-β1 and the downstream signaling pathway in cells directly derived from patients’ wounds.

**Fig. 4:**
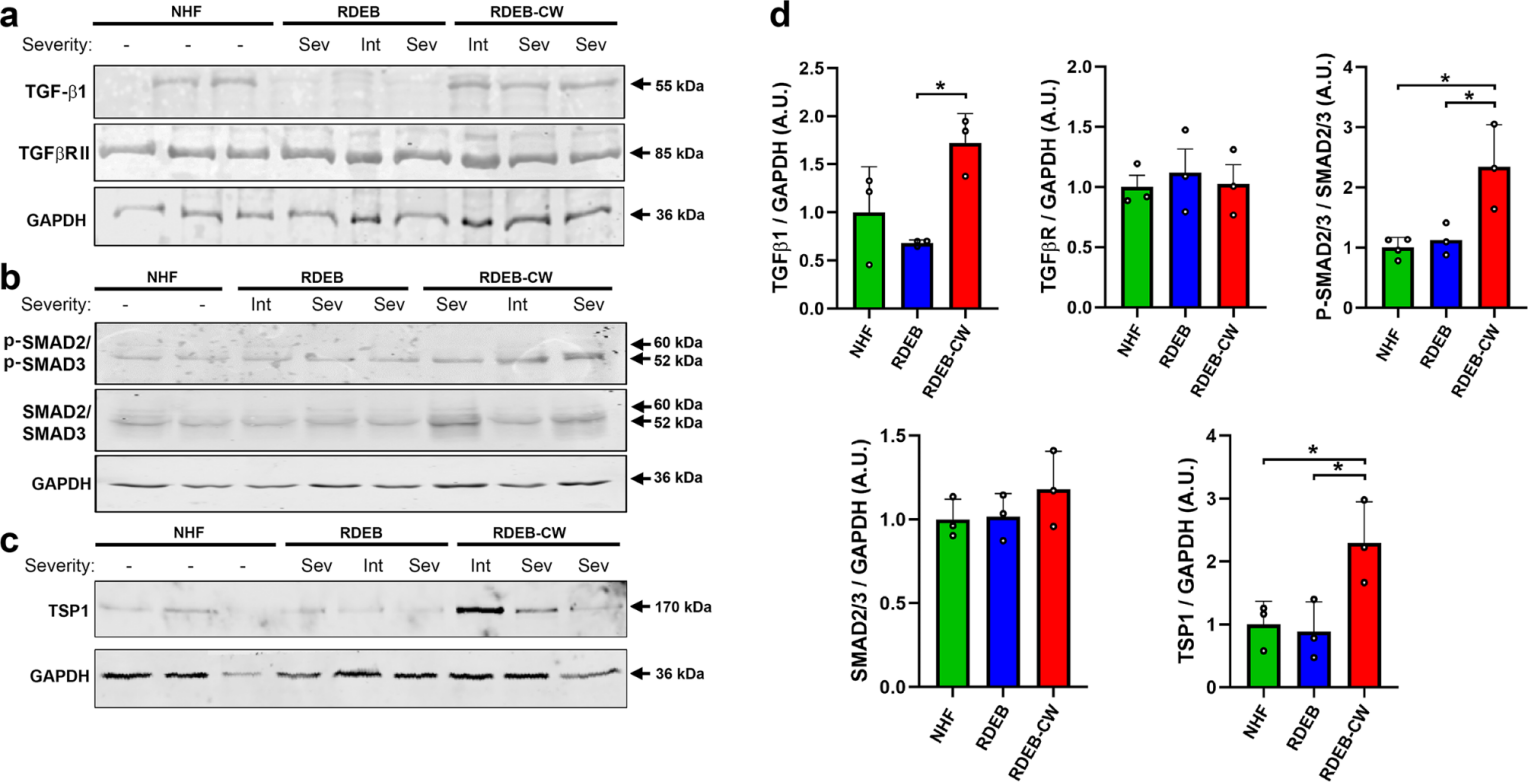
**Analysis of TGF-β signaling in RDEB-CW fibroblasts. (a-c)** Western blot analysis of protein lysates derived from fibroblast cultures to detect the immature form of TGF-β1 and its receptor, TGFβRII **(a)**, the SMAD2/SMAD3 proteins and their phosphorylated forms (p- SMAD2/p-SMAD3) **(b)**, and the TGF-β1 activator molecule, TSP1 **(c)**. **(d)** Densitometric analysis of all western blots displayed in **(a-c)**. All data are expressed as mean ± SD. The experiments were performed with n = 3–4 per condition. Asterisks indicate significant differences by one-way ANOVA with Tukey post-hoc (p < 0.05)

First, we analyzed the expression of proteins related to the TGF-β1 pathway in NHF, RDEB and RDEB-CW fibroblasts, including TGF-β1 and its receptor TGFBR-II, the TGF-β1 downstream effectors SMAD2/3, and the protein activator of TGF-β1, thrombospondin 1 (TSP1) [64]. Our western blot analysis showed an increased abundance of the TGF-β1 precursor protein in RDEB-CW fibroblasts (Fig. 4a, d), which results in the functional growth factor once it has been properly processed [65]. We did not detect significant changes in the TGFBR-II receptor levels (Fig. 4a, d). We next analyzed the abundance and phosphorylation status of SMAD2/SMAD3 proteins (Fig. 4b). We detected a significant increase in the abundance of p-SMAD2/p-SMAD3 proteins in RDEB-CW fibroblasts compared to NHF and RDEB fibroblasts (Fig. 4d), but the levels of total SMAD2/SMAD3 showed no significant differences (Fig. 4b, d), which is consistent with that described for canonical activation of the TGF-β1 pathway [66]. Furthermore, the TGF-β1 activator TSP1 was increased exclusively in RDEB-CW fibroblasts (Fig. 4c, d). Taken together, all these results indicate that the TGF-β1 pathway is highly activated in RDEB-CW fibroblasts, meanwhile RDEB fibroblasts, in our laboratory conditions, showed TGF-β1 activity levels similar to NHF cultures. Interestingly, no differences were observed between protein abundance and patient disease severity, suggesting that fibroblasts isolated from RDEB chronic wounds have common molecular characteristics surpassing the overall patient disease burden.

### Expression of inflammatory cytokines in RDEB-CW fibroblasts

**Fig. 5:**
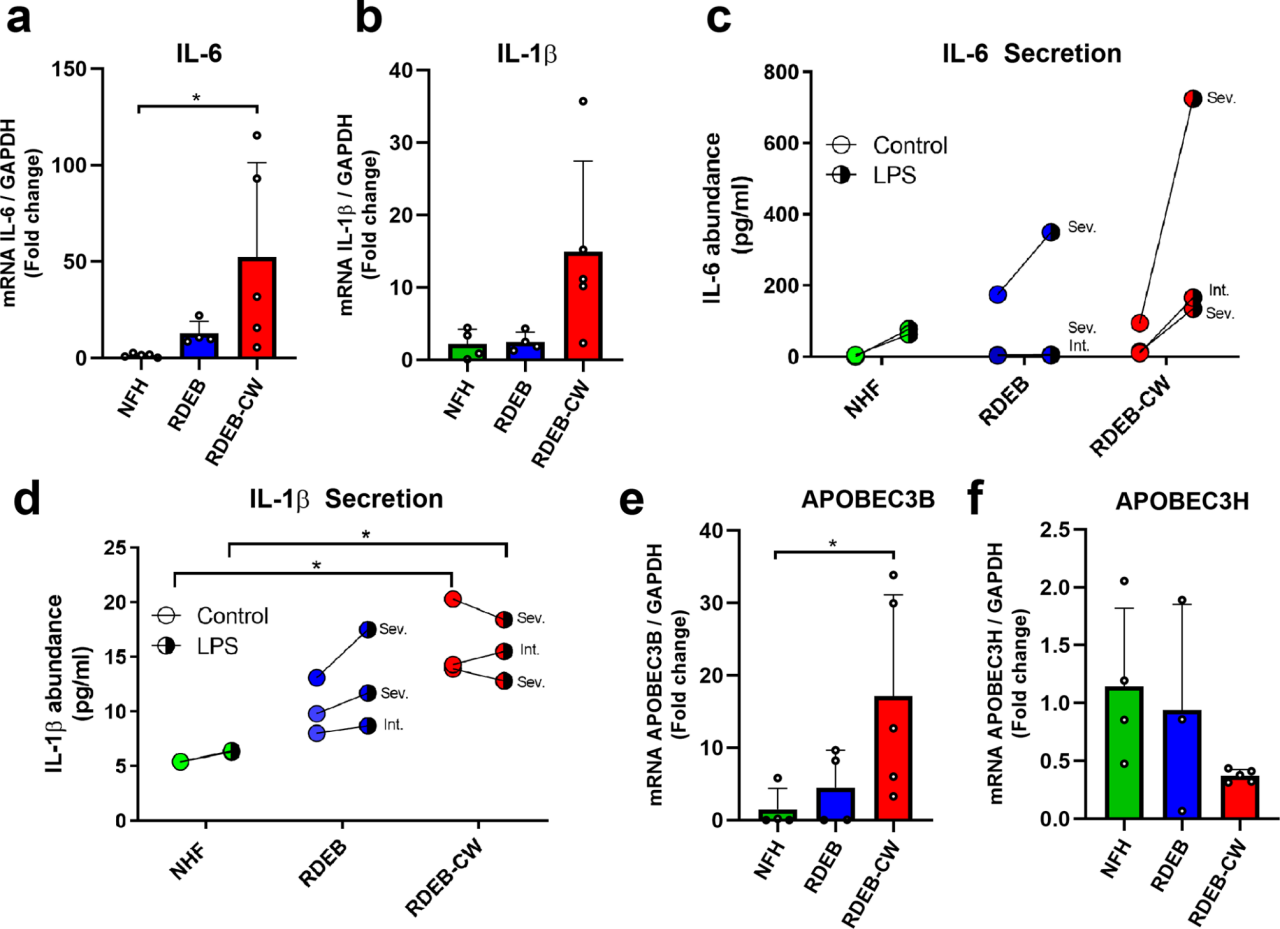
**Expression of inflammatory cytokines and cancer-related proteins in RDEB-CW fibroblasts**. **(a-b)** Reverse Transcription (RT) qPCR analysis of inflammatory cytokines IL-1β and IL-6. Asterisks indicate significant differences by one-way ANOVA with Tukey post-hoc (p < 0.05, n = 4–5). **(c-d)** Cytokine analysis in supernatants of fibroblast cultures by flow cytometry, using the CBA cytokine kit for detecting IL-1β **(c)** and IL-6 **(d)**. Disease severity is indicated for RDEB-derived patient samples (n = 2–3). Asterisks indicate significant differences by Kruskal-Wallis with Dunn’s multiple comparisons test (p < 0.05). **(e-f)** RT-qPCR analysis of two cancer-related genes *APOBEC3B* **(e)** and *APOBEC3H* **(f)**. Asterisks indicate significant differences by one-way ANOVA with Tukey post-hoc (p < 0.05, n = 4–5). All data are expressed as mean ± SD, except for data shown in Figures **(c)** and **(d)**

We analyzed the abundance of two pro-inflammatory cytokines, IL-6 and IL-1β by reverse transcription quantitative PCR (RT-qPCR) analysis and flow cytometry assays simultaneously. We observed a significant increase on IL-6 gene expression in RDEB-CW fibroblasts but also a high degree of variability between samples from different patients (Fig. 5a). This variability was also observed in IL-1β (Fig. 5b); however, no significant increase was found for this cytokine. Next, using a flow cytometry assay, we assessed RDEB-CW cells ability to secrete these cytokines, utilizing lipopolysaccharide (LPS, 1ug/ml) as a positive stimulus for secretion. Similar to our findings by RT-qPCR analysis, IL-6 secretion trended to increase in LPS-activated RDEB-CW fibroblasts (Fig. 5c), although no significant differences were observed between groups. On the other hand, IL-1β secretion was increased in basal and LPS-induced conditions in RDEB-CW fibroblasts compared to NHF fibroblasts (Fig. 5d), meanwhile RDEB fibroblasts showed no significant differences compared to the others groups with or without LPS treatment. Interestingly, our results indicate that RDEB-CW fibroblasts did not show an increase in IL-1β secretion followed LPS treatment, which could indicate the pre-existence of an inflammatory state in these cells (mean of 16.17 ± 2.1pg/ml in control condition, mean of 15.57 ± 1,6pg/ml in LPS treatment; p: 0.83 by Student T-test) (Fig. 5d).

We also evaluated the expression of three genes of the apolipoprotein B mRNA editing enzyme, catalytic polypeptide like type 3 (APOBEC3) family: *APOBEC3A*, *APOBEC3B* and *APOBEC3H*. APOBEC3 proteins are cytosine deaminases known to play a role in viral infection and inflammation [67–69], and these proteins have been shown to drive mutation acquisition in RDEB-associated SCC [69]. Moreover, it has also been associated to the activity of IL-6 [70]. Of these three genes, only *APOBEC3B* was found to have increased expression in RDEB-CW fibroblasts (Fig. 5e). We did not observe significant differences in *APOBEC3H* levels between the experimental groups (Fig. 5f), and no detectable expression of *APOBEC3A* was found in any of the analyzed samples.

## Discussion

### RDEB-CW fibroblasts as an in vitro model of RDEB

Currently, there are limited cellular models that efficiently recreate the physiological and molecular parameters observed in RDEB wound healing in vitro. Nevertheless, primary fibroblasts derived from RDEB patient’s biopsies have been successfully cultured and studied showing the potential to model some of the physiological processes observed in these patients. However, reported data has shown that these cells exhibit a broad range of morphological, migratory and proliferative properties, possibly attributed to differences in laboratory conditions, techniques, patient variability, and others. For example, Beilin et al. revealed that RDEB patient derived fibroblasts displayed an increased area and a more spread-out morphology than NHF [25], and in our results we show that RDEB and NHF presented similar morphological features. Additionally, Chen et al. reported that RDEB patient derived fibroblasts displayed a reduced proliferative potential compared to NHF or to RDEB fibroblasts expressing recombinant C7 [26]. However, in relation to proliferation rates, other authors have shown that RDEB patient’s derived fibroblasts displayed no significant changes compared to NHF [27–29]. Similarly, Condorelli et al. reported that RDEB fibroblasts displayed no significant changes in migration assays in vitro, while Georgiadis et al. demonstrated that RDEB fibroblasts showed impaired migration potential compared to NHF or RDEB expressing recombinant C7 [27, 28]. In our study, we found that RDEB fibroblasts display no significant differences in proliferation nor migration compared to NHFs in vitro. On the other hand, our morphological and functional analysis revealed that RDEB-CW fibroblasts exhibited an increase in cell area and protrusions number, and also an impaired proliferation and migratory rate in vitro, reminiscent of the senescent phenotype observed in chronic wound-derived fibroblasts obtained from patients with venous leg ulcers [71]. Furthermore, we observed that RDEB-CW fibroblasts exhibit an increase in senescence associated β-galactosidase population, and a change in the subcellular localization of the Lamin B1 marker, which have been previously associated to be increased in senescent cells [34, 36, 39]. Previous studies indicated that low-passages primary fibroblast cultures displayed a senescent cell population of about 10% [38], which is consistent with the results we obtained for NHF and RDEB fibroblasts assessed by the senescence associated β-galactosidase marker. A previous study demonstrated a clinical correlation between quantitative in vitro senescence and the time it takes the ulcerous tissue to heal, where populations of senescent fibroblasts over 15% were identified as the threshold beyond which a wound would become difficult to heal [72]. We found that RDEB-CW fibroblasts had a senescent population close to 20%; thus, increased senescence in the fibroblast population could explain some of the defects we observed at the level of proliferation and migration in vitro, and in wound healing in RDEB patients in vivo.

Interestingly, an increased senescent state has been described in cancer-associated fibroblasts (CAFs) [73–75]. CAFs are known to secrete factors contributing to cell viability and modulating cancer progression. Considering that these patients have an increased risk of developing cSCC early in life, this in vitro model could potentially be a valuable tool for diagnostic studies associated to wound microenvironment, and also for the identification of new, early cSCC markers in RDEB patients.

### Advantages and limitations of studying RDEB-CW fibroblasts.

In this work, we showed that fibroblasts cultured from discarded wound dressings maintain several hallmarks of a chronic wound, which could make this model a valuable asset in both research and diagnostic studies. For example, we have shown that these fibroblasts produce more stress fibers and are capable of increased contraction of collagen matrices, which implies a higher fibrotic capability. This in turn could provide a manner to more accurately assess the severity of the wound, as fibrosis is an indicator of wound severity and cSCC risk [76]. The latter extends to several other markers as well, for example, here we studied the expression of three APOBEC3 family proteins, which are known to be implicated in the formation of several types of cancer, including cSCC in RDEB patients [69, 70, 77]. Of note, APOBEC3 expression is likely more in important in keratinocytes, the cell of origin for RDEB cSCC but increases in expression could well represent a surrogate for levels of APOBEC within wounds. In our study, we showed a significantly increased expression of one APOBEC3 subunit (*APOBEC3B*) in RDEB-CW fibroblasts. Our observation, although new requires further work in order to determine its relevance for cSCC development. As cSCC in RDEB patients is a highly aggressive form of cancer, being able to differentiate wounds that develop into SCC would greatly assist early detection and clinical management. In addition to measuring senescence, wound-derived fibroblasts could also be tested for skin age-associated markers, such as the production of reactive oxygen species, telomere length, among others [78]. More importantly, this particular method of culturing cells from wound dressings could not only be used to collect other cell types such as keratinocytes, but could also be expanded to study fibrocytes, macrophages and immune cells through the use of selective culturing and FACS [30]. In addition, this technology could also be beneficial to study wound microenvironment in other, non-EB related diseases, such as pyoderma gangrenosum, ischemic skin lesions, dermatitis, psoriasis, diabetic and venous ulcers, as well as age-related chronic wounds.

Despite the fact that our cell model has shown to have several interesting advantages for studying part of the RDEB physiopathology in vitro, it is necessary to be cautious when comparing cell populations obtained with different culturing strategies. In this study, we morphologically and functionally compared RDEB-CW fibroblasts obtained from discarded wound dressings with fibroblast cultures obtained from skin biopsies from RDEB patients and healthy controls. One limitation of this study is the fact that skin fibroblast populations are heterogeneous. Previous findings have revealed the existence of different fibroblast lineages, each having specific functions according to their localization in the papillary or the reticular dermis [40, 79]. However, these studies have been limited because the lack of specific markers to differentiate between these two populations. Thus, the morphological, functional, and molecular analysis we carried out in this work represent an overview of processes that could be studied in greater detail in the future. Additionally, in this work we focused on characterizing the level of activation/differentiation in low-passage fibroblast cultures, though it would be interesting to analyze whether RDEB-CW fibroblasts can retain this phenotype over time, in the absence of the pro-inflammatory and fibrotic microenvironment from which they were harvested.

Finally, our approach has the potential to reconstruct the history of a particular wound, as cells isolated at different time-points during wound progression could be isolated, characterized and compared. If combined with high throughput methods, these cells could potentially be a powerful tool to study the evolution of a wound from an acute to a chronic status, comparing the molecular signatures, physiological responses, ECM or cytokine secretion, morphological patterns, and others. Such a well-defined, controlled in vitro culture environment could provide a platform for studying mechanisms of wound healing as well as cancer detection and treatment.

## Conclusions

Our results demonstrate that primary fibroblasts isolated from discarded dressings of RDEB chronic wounds recapitulate multiple parameters of wound chronicity, and are a potentially useful research tool to study chronic wounds in vitro. Additional work with a larger sample size would be optimal to further confirm these findings in other RDEB populations with translation into clinical settings.

## Methods

### Cell samples

Adherent cells used in this work are part of the DEBRA Chile cell bank. Skin biopsies, either from healthy individual donors or RDEB patients, were utilized to isolate and culture dermal fibroblasts. Adherent cells isolated from chronic wounds were obtained from discarded wound dressings and cultured as described in [30]. Wounds included in this study were part of a larger longitudinal follow-up study previously published [71]. Chronic wounds were defined as wounds that were opened for more than 1 year at the time of dressing sampling, and remained opened for at least 3 months after. Acute wounds were sampled if present for > 14 days and all 3 acute wounds included in this study closed within 6 weeks of dressing collection. Cells used for the experiments are indicated on Table 1. Written informed consent was obtained from each patient prior to sample collection. This study was approved by the Ethics Committee of Clínica Alemana Universidad del Desarrollo #2013 − 145.

THP-1 cells are a monocyte-like line derived from a leukemia patient (ATCC#TIB-202). Cells were growth in RPMI-1640 medium (Gibco, USA) containing 10% FBS and 2mM L-glutamine (Gibco, USA).

### Cell morphology analysis

Five brightfield images, at a 40–60% confluency, were taken for each cell line. For each image, 15 cells were randomly selected and the area and the protrusions number were measured using the Image J software (NIH, USA). Protrusions were considered to be any cell elongation larger than one third of the cell size, excluding the spindle-like shape commonly seen in fibroblasts. A total of 225 cells per experimental group were used for the quantitative analyses.

### RNA extraction, complimentary DNA synthesis and quantitative PCR

RNA extraction was performed with TRIzol reagent (Invitrogen, USA), following the manufacturer’s guidelines. RNA integrity was confirmed through electrophoresis using a MOPS/Formaldehyde gel. DNA contaminations were eliminated with DNAse I (Thermo Scientific) and complementary DNA (cDNA) was synthesized using the High Capacity cDNA Reverse Transcription kit (Applied Biosystems), according to manufacturer’s protocols.

Quantitative PCR (qPCR) was performed utilizing either the Brilliant III Ultra-Fast SYBR® Green QPCR Master Mix (Agilent Technologies, USA) and the SYBR Select Master Mix (Thermo Fisher Scientific, USA), according to the protocol specified by the manufacturers. The qPCR reactions were carried out in a Stratagene Mx3005P (Agilent Technologies, USA). Data was processed through the 2^-ΔΔCt^ method [80]. Primers used in this study are shown in Supplementary Table 1.

### Indirect Immunofluorescence

Cells were plated on 10 mm coverslips in 24-well plates until they reached ~ 80% confluence. Cells were fixed in 4% formaldehyde solution, and then permeabilized with 0.2% Triton-X100. Next, cells were blocked using a 3% BSA solution. Primary antibodies were incubated overnight at 4 °C, and secondary antibodies were incubated by 2 h at room temperature. Coverslips were mounted into glass slides with Fluoromount G with DAPI (Invitrogen, USA), and then sealed with commercial transparent nail polish. All the antibodies used in this study are listed in Supplementary Table 2.

Pictures were taken with the EVOS FL Cell Imaging System (Thermo Fisher Scientific, USA) or the Fluoview FV10i confocal microscope (Olympus, Japan). Images were analyzed using ImageJ software (NIH, USA).

### Western Blot

Cell lysates were prepared on ice with radio immunoprecipitation assay buffer (RIPA, Cell Signaling Tech., USA) with proteinase inhibitors (Sigma-Aldrich, Merck, USA) and phosphatase inhibitor cocktail (SCBT, USA). Protein quantification was performed using Pierce™ BCA Protein Assay Kit (ThermoFisher, USA). Cell lysates were separated on 7–10% SDS-polyacrylamide gels (SDS-PAGE) and transferred into nitrocellulose membranes (Bio-Rad, USA) via a semi-dry transfer (using Thermo Fisher’s PowerBlotter XL kit). Membranes were blocked with 5% BSA for 1 h at room temperature and then incubated overnight with the primary antibodies at 4 °C. Fluorescent-conjugated secondary antibodies were incubated at room temperature for 1 h. The membranes were revealed using the Odyssey CLx Infrared Imaging System (LI-COR Biosciences, USA). Images were quantified using ImageJ software (NIH, USA). All antibodies used in this study are listed in Supplementary Table 2. Rabbit polyclonal collagen VII antibody raised against the NC1 domain was used thanks to M. Peter Marinkovich, MD, Stanford University School of Medicine, Stanford, USA [81].

### Cell proliferation assay

Fibroblasts were seeded at 1 × 10^4^/cm^2^ density in black, polystyrene 96-well plates in quadruplicate, and a fluorescence-based proliferation assay (CellTiter-Blue® Assay, Promega, USA) was carried out according to manufacturer’s instructions every 24 h over 3 days. Cells were grown in DMEM supplemented with 10% FBS, and 2 h prior to obtaining fluorescence readings, culture medium was replaced by phenol red-free, FBS-free DMEM, and CellTiter-Blue® Reagent was added. Fluorescence was analyzed at 580 nm in a Modulus™ Microplate Multimode Reader (Turner Biosystems, USA).

### 2D migration assay

Fibroblasts were grown in DMEM supplemented with 10% FBS until reaching a confluent monolayer. To register the same fields for each image acquisition, we traced reference lines in the plates with a tip marker. Cell cultures were scratched with a 200 µl sterile pipette tip, and then the plates were washed with PBS to discard detached cells. Transmitted light camera images were obtained immediately after scratch induction, and then after 8 and 24 h in culture. We determined the wound area using the method and Image J plugin developed by Suarez-Arnedo et al. [82]. Results were expressed as mean wound area (% of initial wound area).

### Collagen disc contraction assay

Cell contractility assays were carried out according to protocol described by Ngo et al. [83], as floating matrices [84]. Briefly, 100,000 fibroblasts were seeded in a type-I collagen solution (rat tail Collagen I, sc-136,157, SCBT, USA) diluted in DMEM (collagen-I in a final concentration of 1 mg/ml) supplemented with 10% FBS, and collagen matrix was allowed to polymerize by adding 2 µl NaOH 1 M (500 µl of cell suspension per well in 24-well plates). Then, 400 µl of complete culture medium was added to each well, and the gel was gently dissociated from the plate using a 200 µl pipette tip. The gel contraction was documented through capturing daily digital images for 3 days, and results were expressed as mean disc area (% of initial disc area).

### Senescence-associated β-galactosidase staining

Senescence-associated β-galactosidase staining kit (Cell signaling, USA) was carried out according to manufacturer’s instructions. Briefly, fibroblasts were grown in DMEM supplemented with 10% FBS until reaching a confluence of 80–90%. Then, plates were rinsed with PBS and fixed, and the β-Galactosidase Staining Solution (pH 6,0) was added. Plates were sealed and incubated overnight in a dry incubator (37° C). Transmitted light camera images were obtained immediately after incubation. We determined the percentage of β-galactosidase positive cells (~ 4000 cells per condition) and the cell area (50 cells per condition) from 20 random fields per condition (4X objective).

### Quantification of subcellular distribution of lamin B1

In order to quantify the subcellular distribution of LaminB1, we obtained fluorescence images of fibroblast cultures stained with LaminB1 and co-stained with DAPI (~ 150 cells per experimental group were analyzed). The cells were classified into four different conditions for further analysis: nuclear distribution, nuclear and cytoplasmatic distribution, cytoplasmatic distribution or none detected. A χ^2^ analysis was carried out to compare the distribution of Lamin B1 subcellular localization between the different experimental groups.

### Cytokine analysis in supernatant by flow cytometry

Fifty thousand fibroblasts were seeded in DMEM with 10% FBS and incubated for 24 h. Afterwards, the media was replaced by phenol red-free and FBS-free DMEM with or without LPS 1 ug/ml and incubated for 24 h before collecting the supernatants. IL-6 and IL-1β levels in the supernatant were measured by the BD™ FACSCanto Flow Cytometer and BD™ Cytometric Bead Array (CBA) Human Inflammatory Cytokines Kit (BD Biosciences, USA). The detailed experiment procedure was conducted according to the instruction manuals of the kit and the flow cytometer. The sensitivity for IL-6 and IL-1β measurement were 3.6 pg/ml and 7.1 pg/ml in this assay, respectively.

### Statistical analysis

Quantitative data were presented as mean ± S.D. Comparisons between groups, plot design and statistical analysis were performed with GraphPad Prism 7.0 (GraphPad Software Inc., USA). Significant differences were analyzed by one-way ANOVA with a Tukey post-hoc, unless another analysis has been indicated for a specific assay. A p value < 0.05 was considered significantly different.

## Electronic supplementary material

Below is the link to the electronic supplementary material.

Supplementary Material 1

Supplementary Material 2

Supplementary Material 3

Supplementary Material 4

Supplementary Material 5

Supplementary Material 6

Supplementary Material 7

## Data Availability

All data generated or analyzed during this study are included in this published article and its supplementary information files.

## List of abbreviations

α-SMA: alpha-smooth muscle actin
APOBEC3: apolipoprotein B mRNA editing enzyme, catalytic polypeptide like type 3
C1: type I collagen
C7: type VII collagen
CAF: cancer-associated fibroblasts
cSCC: cutaneous squamous cell carcinoma
ECM: extracellular matrix
DEB: Dystrophic Epidermolysis Bullosa
EB: Epidermolysis Bullosa
EBS: Epidermolysis Bullosa simplex
FAP: fibroblast activating protein
IIF: indirect immunofluorescence
IL-1: interleukin-1
IL-6: interleukin-6
JEB: Junctional Epidermolysis Bullosa
KEB: Kindler Epidermolysis Bullosa
KGF: keratinocyte growth factor
LPS: Lipopolysaccharide
MSC: Mesenchymal stem cells
NHF: normal human fibroblasts
RDEB: Recessive Dystrophic Epidermolysis Bullosa
RDEB-AW: Recessive Dystrophic Epidermolysis Bullosa acute wounds
RDEB-CW: Recessive Dystrophic Epidermolysis Bullosa chronic wounds
TGF-: Transforming Growth Factor-
TSP1: thrombospondin 1
WB: western blot
