# Supplementary material for: Maintenance of chronicity signatures in fibroblasts isolated from recessive dystrophic epidermolysis bullosa chronic wound dressings under culture conditions
Source: Biol Res. 2023 May 10;56:23. doi: 10.1186/s40659-023-00437-2 (PMC10170710; doi:10.1186/s40659-023-00437-2)
Supplement: Supplementary file 1 — Supplementary Material 1 [file 40659_2023_437_MOESM1_ESM.docx]

**
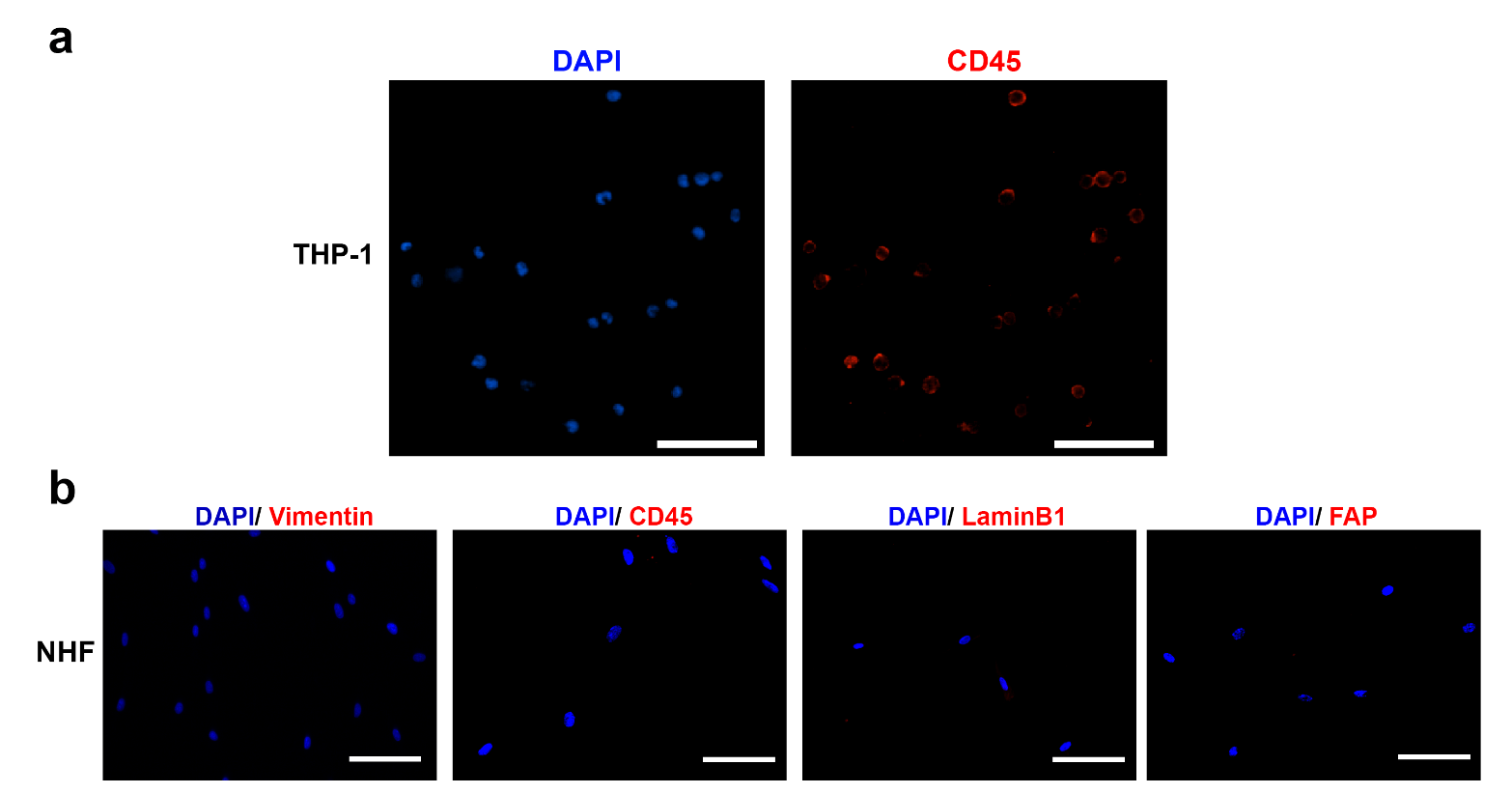
**

**Supplementary Figure 1. Standardization of indirect immunofluorescence cell markers. (a)** Representative fluorescence images for THP-1 cells (a human monocytic cell line, as a positive control for CD45 expression) stained with the CD45 marker (red), and co-stained with DAPI (Blue). Bar: 100μm. **(b)** Fluorescence microscopy images obtained for standardizing the cell marker antibodies. To rule out non-specific signals or autofluorescence of the secondary antibodies, staining of NHF cultures was performed without the primary antibodies. DAPI staining allows the identification of cell nuclei in each condition. Bar: 100μm.
