# Supplementary material for: Maintenance of chronicity signatures in fibroblasts isolated from recessive dystrophic epidermolysis bullosa chronic wound dressings under culture conditions
Source: Biol Res. 2023 May 10;56:23. doi: 10.1186/s40659-023-00437-2 (PMC10170710; doi:10.1186/s40659-023-00437-2)
Supplement: Supplementary file 2 — Supplementary Material 2 [file 40659_2023_437_MOESM2_ESM.docx]

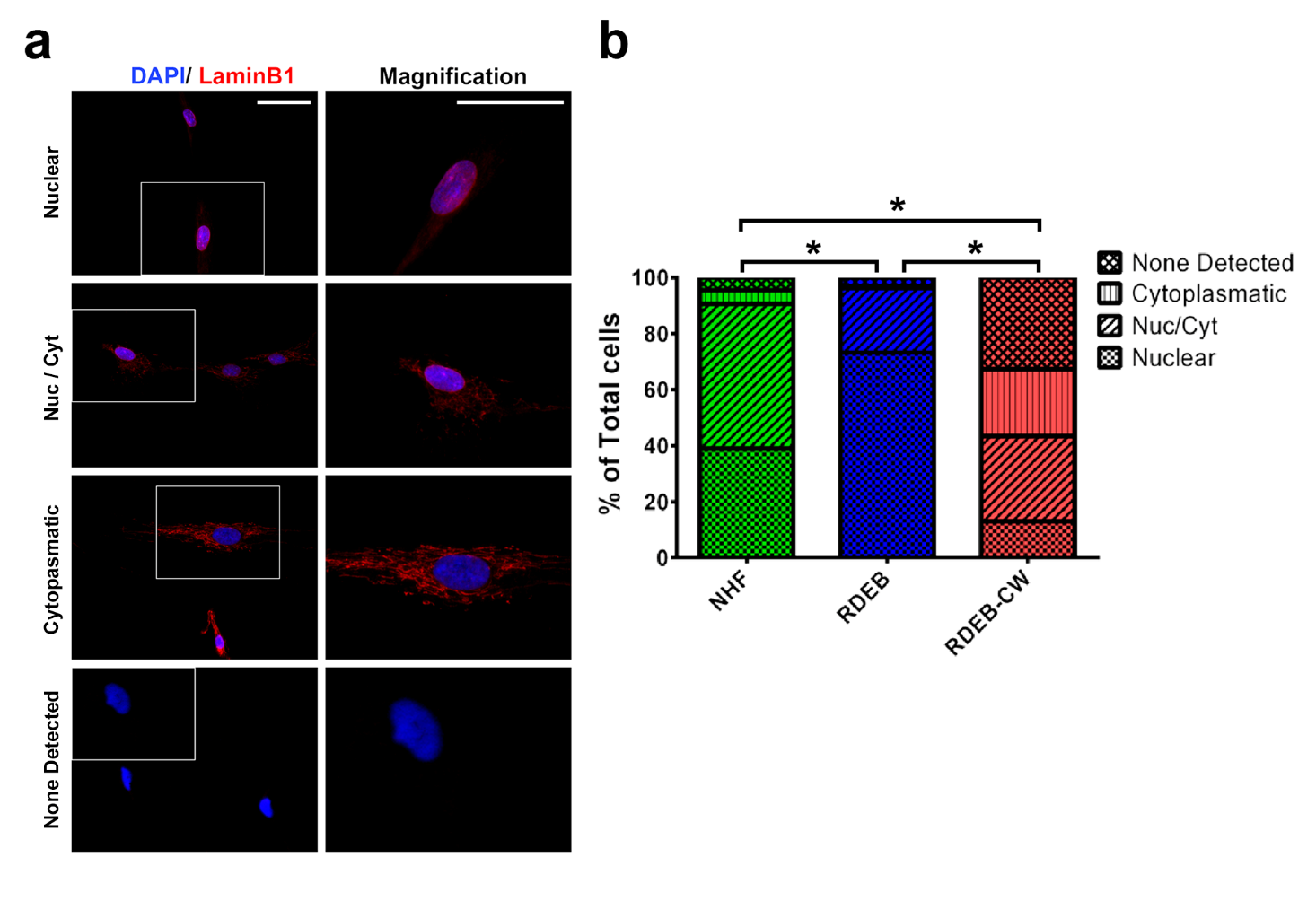


**Supplementary Figure 2. Subcellular distribution of Lamin B1. (a)** Representative fluorescence images illustrating the subcellular distribution of Lamin B1(red) in fibroblast cultures. Cells were co- stained with DAPI (Blue). Each cell was classified according to their Lamin B1 subcellular distribution, recognizing a nuclear, nuclear and cytoplasmatic (Nuc/Cyt), cytoplasmatic or none detected localization. Bar: 100 µm. **(b)** Stacked bar plot showing the Lamin B1 cell distribution between the three experimental groups. χ2 analysis indicates significant differences (*) for Lamin B1 nuclear distribution between NHF and RDEB, RDEB and RDEB-CW and NHF and RDEB-CW groups (p<0.001). Fifty images of each sample were analyzed (n=3 per experimental group).
