# Supplementary material for: Maintenance of chronicity signatures in fibroblasts isolated from recessive dystrophic epidermolysis bullosa chronic wound dressings under culture conditions
Source: Biol Res. 2023 May 10;56:23. doi: 10.1186/s40659-023-00437-2 (PMC10170710; doi:10.1186/s40659-023-00437-2)
Supplement: Supplementary file 3 — Supplementary Material 3 [file 40659_2023_437_MOESM3_ESM.docx]

**
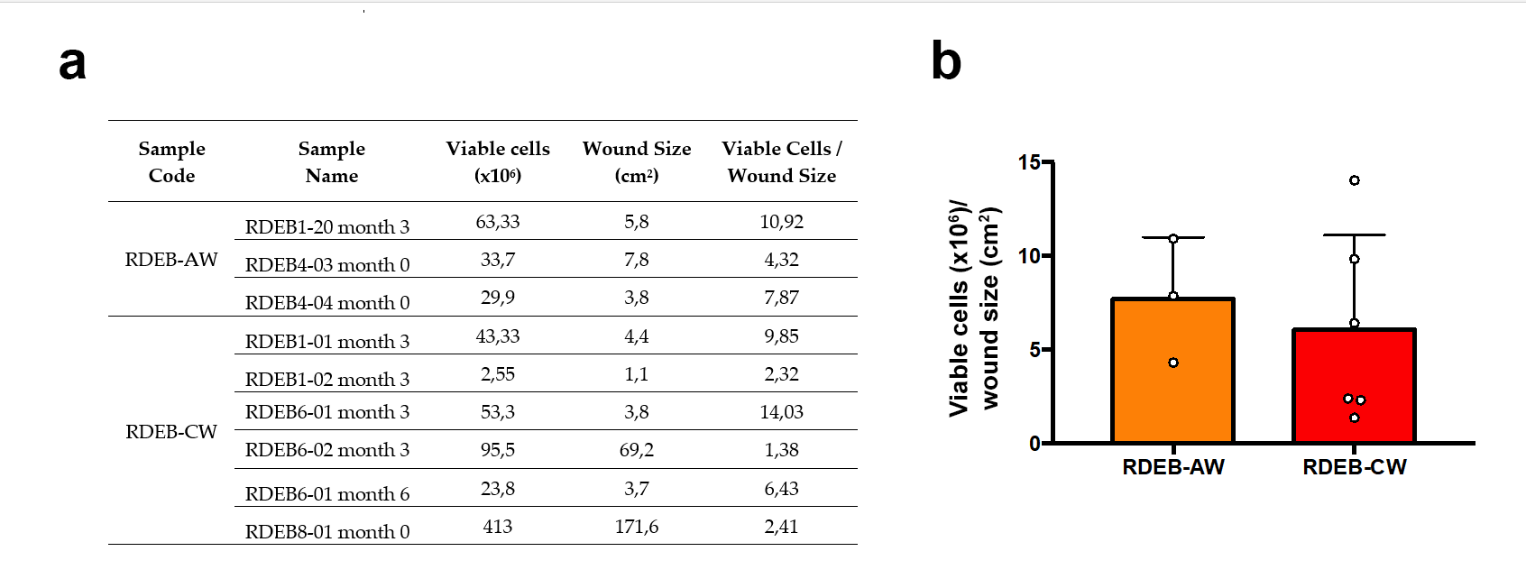
**

**Supplementary Figure 3. Viable cells isolated from wound dressings. (a)** Table showing all wound samples used in this study. **(b)** Graphical representation of the number of viable cells per wound size shown in **(a)**. Cell count and viability was assessed with trypan blue staining by using a hemocytometer as shown previously [31]. Data are presented as mean ± SD, and experimental groups were compared by a student T-test. No significant differences were observed.
