# Supplementary material for: Maintenance of chronicity signatures in fibroblasts isolated from recessive dystrophic epidermolysis bullosa chronic wound dressings under culture conditions
Source: Biol Res. 2023 May 10;56:23. doi: 10.1186/s40659-023-00437-2 (PMC10170710; doi:10.1186/s40659-023-00437-2)
Supplement: Supplementary file 4 — Supplementary Material 4 [file 40659_2023_437_MOESM4_ESM.docx]

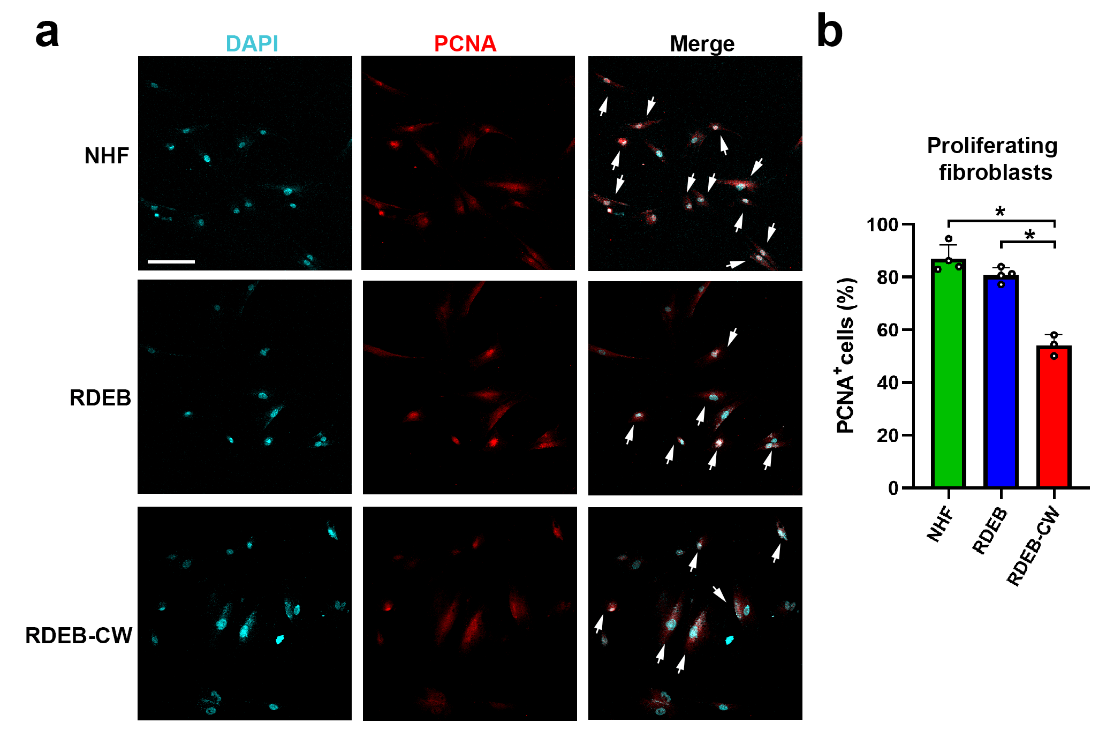


**Supplementary Figure 4: RDEB-CW fibroblasts exhibit a decreased proliferating population. (a)** Representative confocal images of fibroblast cultures stained with the PCNA antibody (red). Nuclei were co-stained with DAPI (cyan). White arrows indicate PCNA positive cells. Bar: 100 µm. **(b)** Quantification of proliferating cells. Data are presented as mean percentage of PCNA^+^ cells± S.D. (n =3-4, one-way ANOVA with Tukey post-test). Asterisks indicate significant differences (p<0.05). A total of ~ 500 cells per culture sample were analyzed, from 20 random fields (10X objective).
