# Supplementary material for: Maintenance of chronicity signatures in fibroblasts isolated from recessive dystrophic epidermolysis bullosa chronic wound dressings under culture conditions
Source: Biol Res. 2023 May 10;56:23. doi: 10.1186/s40659-023-00437-2 (PMC10170710; doi:10.1186/s40659-023-00437-2)
Supplement: Supplementary file 5 — Supplementary Material 5 [file 40659_2023_437_MOESM5_ESM.docx]

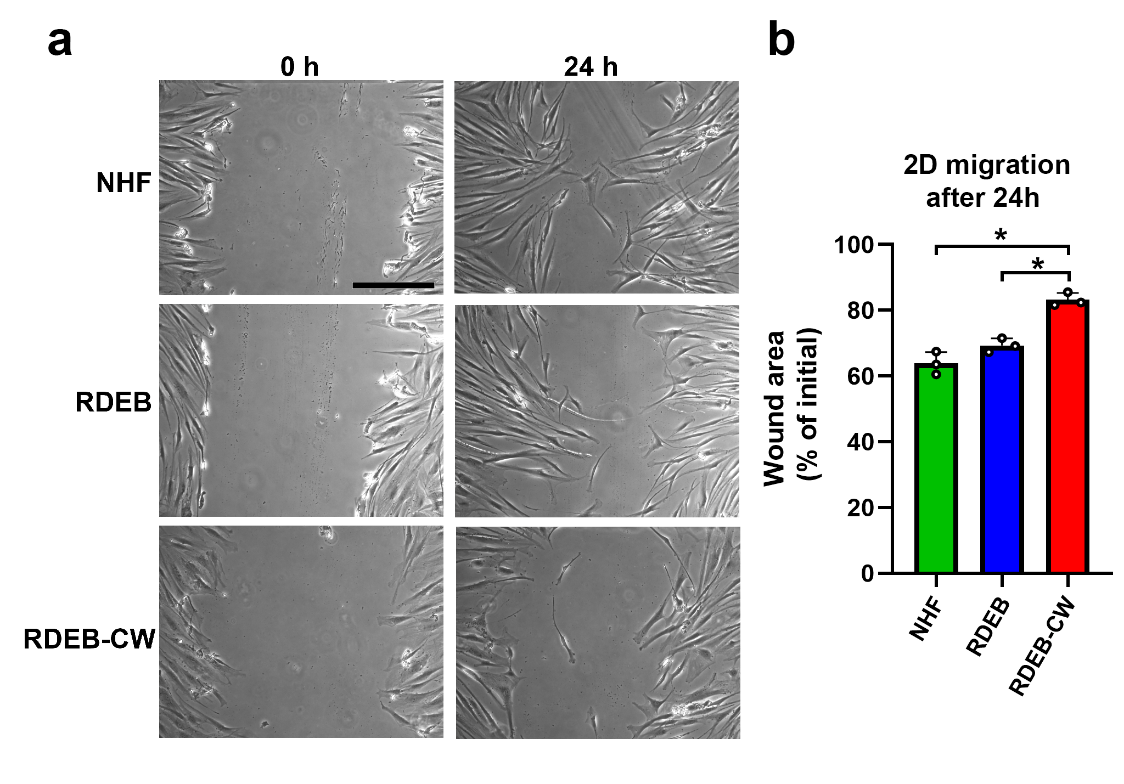


**Supplementary Figure 5. RDEB-CW fibroblast exhibited a reduced migration rate in low serum conditions.** **(a)** Representative brightfield images of 2D migration assays for fibroblast cultures grown in low serum conditions (0.5% FBS), and pre-treated with an anti-mitotic agent (5 µM ARAC, administered 24 hours previous to scratch) Bar: 100 µm. **(b)** Quantification of migration rate, expressed as the percentage of the initial wound area after 24 h *in vitro*. The results are expressed as mean ± SD, and asterisks indicate significant differences by a one-way ANOVA with a Tukey post-hoc (n=3).
