# Supplementary material for: Maintenance of chronicity signatures in fibroblasts isolated from recessive dystrophic epidermolysis bullosa chronic wound dressings under culture conditions
Source: Biol Res. 2023 May 10;56:23. doi: 10.1186/s40659-023-00437-2 (PMC10170710; doi:10.1186/s40659-023-00437-2)
Supplement: Supplementary file 6 — Supplementary Material 6 [file 40659_2023_437_MOESM6_ESM.docx]

| **Gene** | **Forward sequence (5’-3’)** | **Reverse sequence (5’-3’)** | **Amplicon size (bp)** | **Amplified exons** |
| --- | --- | --- | --- | --- |
| *APOBEC3A* | ATGGCATTGGAAGGCATAAG | CAAAGAAGGAACCAGGTCCA | 176 | 3 - 4 |
| *APOBEC3B* | TTCGAGGCCAGGTGTATTTCA | CAGAGATGGTCAGGGTGACA | 195 | 6 – 7 |
| *APOBEC3H* | CCCGCCTGTACTACCACTGG | GGGTTGAAGGAAAGCGGTTT | 152 | 3 - 4 |
| *IL-1B* | CCCAAAGAAGAAGATGGAAAAGC | TCTGCTTGAGAGGTGCTGATG | 114 | 7 |
| *IL-6* | *AGTTCCTGCAGAAAAAGGCA* | *AAAGCTGCGCAGAATGAGAT* | *140* | *5 – 6* |
| *GAPDH* | GGCCTCCAAGGAGTAAGACC | AGGGGTCTACATGGCAACTG | 147 | 9 |

**Supplementary Table 1.** qPCR primers used in this study.
