# Supplementary material for: Maintenance of chronicity signatures in fibroblasts isolated from recessive dystrophic epidermolysis bullosa chronic wound dressings under culture conditions
Source: Biol Res. 2023 May 10;56:23. doi: 10.1186/s40659-023-00437-2 (PMC10170710; doi:10.1186/s40659-023-00437-2)
Supplement: Supplementary file 7 — Supplementary Material 7 [file 40659_2023_437_MOESM7_ESM.docx]

**Supplementary Table 2.** List of antibodies used for this study.

| **Primary antibodies** | | | | |
| --- | --- | --- | --- | --- |
| **Antibody** | **Catalog Nº** | **Manufacturer** | **Dilution used in IF** | **Dilution used in WB** |
| αSMA | MA5-11547 | Invitrogen | 1:200 | 1:1000 |
| CD45 | 304002 | Biolegend | 1:100 | - |
| Collagen I | ab34710 | Abcam | 1:400 | - |
| Collagen I | 91144 | Cell Signaling Technology | - | 1:1000 |
| Collagen VII * | - | - | - | 1:1000 |
| Vimentin | M0725 | Dako | 1:100 | - |
| FAP | sc-65398 | Santa Cruz Biotechnology | 1:100 | - |
| TGF-β1 | 3709 | Cell Signaling Technology | - | 1:1000 |
| TGF-βRII | E5M6F | Cell Signaling Technology |  | 1:1000 |
| YKL-40 | 47066 | Cell Signaling Technology | - | 1:1000 |
| Actin | A5441 | Sigma Aldritch | - | 1:20000 |
| Lamin B1 | 13435 | Cell Signaling Technology | 1:80 | - |
| α-Tubulin | 3873 | Cell Signaling Technology | - | 1:10000/1:20000 |
| Trombospondin-1 | 37879 | Cell Signaling Technology | - | 1:1000 |
| SMAD2/3 | 8685 | Cell Signaling Technology | - | 1:1000 |
| p-SMAD2/3 | D27F4 | Cell Signaling Technology |  | 1:1000 |
| PCNA | NB 600-1331 | Novus Biologicals | 1:100 |  |
| GAPDH | sc-32233 | Santa Cruz Biotechnology | - | 1:500 |
| **Secondary antibodies** | | | | |
| **Antibody** | **Catalog Nº** | **Manufacturer** | **Dilution used in IF** | **Dilution used in WB** |
| Anti-mouse 488 | A11029 | Thermo Fisher Scientific | 1:200 | - |
| Anti-mouse 594 | 405326 | Biolegend | 1:200 | - |
| Anti-mouse 680 | A32729 | Thermo Fisher Scientific | - | 1:10000 |
| Anti-rabbit 488 | A11008 | Thermo Fisher Scientific | 1:200 | - |
| Anti-rabbit 800 | A32735 | Thermo Fisher Scientific | - | 1:10000 |
| Anti-rabbit HRP | 31460 | Thermo Fisher Scientific | - | 1:5000 |

* Collagen VII antibody is a Rabbit polyclonal raised against the NC1 domain, gently donated by M. Peter Marinkovich, MD, Stanford University School of Medicine, Stanford, USA [82].
